# Swallowing, nutritional status, and salivary flow in patients after head and neck cancer treatment, a pilot study

**DOI:** 10.1038/s41598-021-99208-w

**Published:** 2021-10-12

**Authors:** Mariana Inri de Carvalho, Marina Gatti, Renata Ligia Vieira Guedes, Renata Camilla Favarin Froes, Danila Rodrigues Costa, Jhonatan da Silva Vitor, Paulo Sérgio da Silva Santos, Giédre Berretin-Felix

**Affiliations:** 1 Department of Speech Therapy, Bauru School of Dentistry, University of São Paulo, Bauru, SP Brazil; 2 Department of Oral Pathology, Bauru School of Dentistry, University of São Paulo, Bauru, SP Brazil; 3 Department of Speech and Language Pathology, Faculdades Metropolitanas Unidas/FMU, São Paulo, SP Brazil

**Keywords:** Cancer, Oncology

## Abstract

Determine the relationship between swallowing function, nutritional status, and salivary flow in patients after head and neck cancer treatment. This pilot study included 17 patients. Swallowing was assessed through videofluoroscopy and surface electromyography (sEMG), nutritional status through anthropometry and dietary assessment, and salivary flow both with and without mechanical stimulation. Test analysis showed that 66.7% of patients had functional limitations in swallowing in 58.3%, 66.7%, and 58.3% residue scale with an average of a line of barium on a structure for pudding, honey, and liquid consistencies, respectively. Laryngeal penetration was found in 8.3% during the swallowing of liquid. Surface electromyography (sEMG) showed above normal values for muscle activity time during the swallowing of pudding. Anthropometric assessment and muscle and adipose tissue indicated eutrophy. Salivary flow test with mechanical stimulus showed that 82.3% of patients' salivary production was well below the appropriate level. There was a significant correlation between muscle tissue reserve and muscle activity time during swallowing in the studied muscles (left masseter *p* = 0.003, right masseter *p* = 0.001, suprahyoid *p* = 0.001, orbicularis oris = 0.020), all in pudding consistency. This pilot study confirmed the relationship between swallowing and nutritional status for its participants, showing that appropriate protein intake influences muscle activity during swallowing in head and neck cancer survivors.

## Introduction

According to the Brazilian National Cancer Institute, cancer is a disease that impacts millions of people worldwide and is considered a major "public health issue" (INCA, 2020. p. 25)^1^. Head and neck cancer (HNC) is among the various types of cancer, the global impact of which involves about 600,000 people every year^1,2^.

Head and neck cancer includes tumors in the labia, oral cavity, pharynx, larynx, and nasal cavity. It has a multifactorial etiology, which means both genetic and environmental factors cause it. Risk factors are related to smoking, drinking, human viral papillomavirus (HPV) infections, Epstein-Barr virus (EBV) infections, exposure to solar radiation, and carcinogenic agents^3,4^.

Treatment is complex and involves several procedure options such as radiotherapy, chemotherapy, and surgery, which can be performed together or separately^5^.

HNC treatments can cause major temporary and permanent adverse effects, such as dysphagia, xerostomia and hyposalivation, odynophagia, trismus, fatigue, oral mucositis, and altered taste and smell^6–8^. Nutritional impact symptoms can be permanent^3,9^ if not assessed and can even negatively influence head and neck cancer survivors in the long term^10–13^.

Decreased swallowing function can lead to altered nutritional status, which can lead to weight loss and altered muscle mass^13,14^, due to the tumor itself or as a result of the treatment^15^. Such factors harm patients' prognosis, affecting their quality of life, delaying or interrupting their treatment, and increasing morbidity and mortality, in addition to the impact they have on the function of the muscles involved in swallowing^16,17^.

Xerostomia and hyposalivation are also extremely harmful as saliva is essential to bolus formation, and decreased salivary flow can negatively impact both such formation and food swallowing^18,19^.

Surface electromyography (sEMG) can be used to measure the electrical activity of certain muscles. It is a non-invasive method that captures electrical signals using surface electrodes, making it possible to provide quantitative data for diagnosis^20^.

The literature points to the adverse effects of head and neck cancer treatment, such as dysphagia and hyposalivation, which harm patients' nutritional status. However, few studies address such correlation, especially after a long period following treatment completion. Thus, this research aimed to verify the relation between swallowing function, nutritional status, and salivary flow in patients after head and neck cancer treatment.

## Results

In March 2017, all medical records of patients with a history of HNC, who did not have any neurological diseases or current cancer diagnosis in any body location and were undergoing dental treatment at a university research center, with no nutritional monitoring, were selected. Then, 36 patients were recruited, but 10 had already died or showed no interest in participating. Thus, 26 patients agreed to participate, of which 8 abandoned the research and 1 had a cancer recurrence. Therefore, the research included 17 participants, of which 14 are male (82.3%) and 3 female (17.7%), with a minimum age of 30 years and a maximum of 63 years, average of 53 years (SD = 9.3, mean = 58), 64.7% adults (35–59.9 years) and 35.3% elderly (60–78.9 years). These data can be found in Table 1.

**Table 1:** Individual data from the sample after head and neck cancer treatment regarding their gender, mean age, anatomical region, and treatment.

Patient	Gender	Age (years)	Anatomical region	Irradiated region	Treatment	
					Modality	Period after treatment (months)
1	F	46	Upper lip	Lip	SR + RT	13
2	M	44	Nasopharynx	Cervical region	RT	8
3	M	56	Larynx	Larynx	RT + CT	64
4	M	51	Base of tongue	Base of tongue	SR	22
5	M	48	Tonsil	Tonsil	RT + CT	44
6	M	41	Oropharynx	Cervical region	RT + CT	5
7	M	58	Epiglottis	Cervical region	SR + RT + CT	12
8	M	58	Oropharynx	Cervical Region	RT + CT	6
9	F	48	Oral mucosa	Cervical region	SR + RT	4
10	M	30	Soft palate	Cervical region	SR + RT + CT	3
11	M	60	Base of tongue and tonsillar pillar	Base of tongue	SR + RT + CT	32
12	M	62	Base of tongue	Cervical region	RT + CT	14
13	M	61	Base of tongue	Cervical region	RT + CT	10
14	M	61	Larynx	Cervical region	RT + CT	6
15	M	59	Tongue	Tongue	SR	17
16	M	63	Base of tongue	Cervical region	SR + RT + CT	13
17	F	62	Oropharynx	Oropharynx	SR + RT + CT	36
N = 17	M = 14 F = 3	Average = 52	–	–	–	Average = 12 months

*M* male, *F* female, *SR* surgery, *RT* radiotherapy, *CT* chemotherapy.

The participants in this research had cancer in the following anatomical regions: amygdala (CID.C09), tongue base (CID.C01), epiglottis (CID.C10.1), lip (CID.C00), larynx (ICD.C32), oral mucosa (CID.C06.0), nasopharynx (ICD.C11), oropharynx (ICD.C10), and soft palate (ICD.C05.1). These pieces of information were gathered from the patients' medical records at the University of São Paulo's Bauru School of Dentistry (CPC/FOB) clinical research center and classified according to the International Classification of Diseases, 10th Revision (ICD-10).

Most patients (29.4%) had base of tongue cancer (5). Of these, 41.1% (7) underwent radiotherapy and chemotherapy, and 29.4% had surgery with radiotherapy and chemotherapy, 11.8% (2) only had surgery, 11.8% (2) had surgery and underwent radiotherapy, and 5.9% (1) only underwent radiotherapy. Regarding the end of treatment, 76.5% finished in 24 months, 29.4% (5) in 3–6 months, 17.7% (3) in 7 months–1 year, 29.4% (5) between 1 and 2 years, and the minority (23.5%, or 4) ended treatment after more than 2 years.

### Evaluation of salivary flow: results

In the classification of salivary flow with and without mechanical stimulus, most assessed patients had a very low level. In classification with mechanical stimulus, 82.30% (n = 14) had a very low level, 5.90% (n = 1) low level, and 11.80% (n = 2) within the normal standards. In classification without mechanical stimulus, 64.8% (n = 11) had a very low level, 17.6% (n = 3) low level, and 17.6% (n = 3) within the normal standards.

### Videofluoroscopy results

Out of the 17 participants in this research, 14 underwent the videofluoroscopic swallowing exam; however, only 12 exams were possible to analyze as there were recording issues in two cases.

Regarding the degree of dysphagia, according to the *Dysphagia Outcome and Severity Scale (*DOSS) scale, most participants (66.7%, n = 8) had functional swallowing, 25% (n = 3) mild dysphagia, and 8.3% (n = 1) mild to moderate dysphagia.

The assessment of the residue scale proposed by Hind et al.^21^ showed that in the five structures evaluated, most patients had one line of barium in liquid (58.4%, n = 7), honey (66.7%, n = 8), and pudding (58.4%, n = 7) consistencies.

Concerning the assessed structures, most individuals had a score of 1 (one line of barium) in liquid 41.7% (n = 5) and pudding 58.4% (n = 7) consistencies in 2 structures. Finally, 41.7% (n = 5) were found in honey consistency in 3 structures, 80% (n = 4) of which scored 2 (grouping of a line of barium on the structures). No patients had residues in 4 or more structures.

Regarding the Penetration-Aspiration Scale^22^, no patient had laryngeal penetration or laryngotracheal aspiration (material does not enter the airway) in the pudding and honey consistencies. Only 8.3% (n = 1) had laryngeal penetration (material enters the airway, remains above the vocal folds, and then is ejected) for liquid consistency.

### Electromyography results

The results for swallowing food of different consistencies show that both the suprahyoid muscle and the upper orbicularis oris muscle had a similar percentage of electrical activity. However, the orbicularis oris muscle showed decreased swallowing time when compared to the other muscles studied. These data can be found in Table 2.

**Table 2:** sEMG results regarding mean peak and mean contractions (in root mean square—RMS), percentage of electrical activity, and muscle recruitment time during swallowing for pudding consistency.

	Peak (µv)	Mean (µv)	%	Time (s)
**Pudding consistency**				
Left masseter muscle	87.32 ± 7.95	21.72 ± 2.25	30.89	8.96 ± 1.37
Right masseter muscle	72.94 ± 6.81	19.18 ± 2.15	31.12	9.10 ± 1.35
Suprahyoid muscles	89.01 ± 2.19	34.26 ± 1.86	40.14	8.89 ± 1.43
Orbicularis oris	87.66 ± 5.20	29.95 ± 2.01	39.14	7.07 ± 1.38
**Honey consistency**				
Left masseter muscle	66.33 ± 3.87	19.20 ± 1.59	34.71	5.37 ± 0.62
Right masseter muscle	54.54 ± 2.16	16.57 ± 0.81	35.99	5.51 ± 0.65
Suprahyoid muscles	76.85 ± 3.94	32.67 ± 2.12	45.36	6.00 ± 0.80
Orbicularis oris	78.55 ± 9.40	31.76 ± 3.61	45.78	4.72 ± 0.49
**Liquid consistency**				
Left masseter muscle	50.63 ± 4.23	20.37 ± 2.09	45.68	2.78 ± 0.28
Right masseter muscle	36.17 ± 2.70	15.11 ± 1.60	47.35	2.93 ± 0.34
Suprahyoid muscles	70.77 ± 4.28	33.88 ± 1.19	50.89	2.46 ± 0.26
Orbicularis oris	64.49 ± 7.63	30.56 ± 3.02	49.63	1.83 ± 0.31

Descriptive analysis.

% = percentage.

### Anthropometric assessment of the nutritional status: results

The anthropometry results showed that 41.20% (n = 7) of the assessed patients were overweight, 35.30% (n = 6) were classified as eutrophic, and 23.50% (n = 4) were underweight according to their body mass index (BMI).

Regarding the arm muscle circumference (AMC), 52.9% (n = 9) of patients were classified as eutrophy, 23.5% (n = 4) as mild malnutrition, 11.8% (n = 2) as moderate malnutrition, and 11.8% (n = 2) as severe malnutrition. Besides, 23.50% (n = 4) of partients had very low, 17.60% (n = 3) had very good, and 17.60% (n = 3) had very high body fat percentage (%BF).

About body fat percentage, 41.2% (n = 7) had levels within normal standards, 23.5% (n = 4) were well below, and 35.3% (n = 6) were above normal standards.

### Dietary assessment of food consumption: results

Dietary assessment found that patients consume more calories than they need, but their macronutrient distribution is within normal standards even though most of them experience vitamin and mineral deficiency. The results showed that 64.70% (n = 11) of patients throughout the day consumed an amount of energy greater than the recommended by ASPEN^23^. Regarding macronutrient distribution, 29.40% (n = 5) of lipid consumption was above the needs recommended by the DRIs^24^ (Table 3).

**Table 3:** Consumption mean and standard deviation, percentage, and number of patients classified according to the need for energy, carbohydrate, protein, and lipid variables according to ASPEN^23^ and micronutrients according to the DRIs^24^.

Nutrients	Mean ± SD	Classification		
		Below	Appropriate	Above
Energy (Kcal)	2453 ± 1001	29.40% (5)	5.90% (91)	64.7% (11)
Carbohydrate (g)	48.66 ± 14.4	23.50% (4)	70.60% (12)	5.9% (1)
Protein (g)	21.33 ± 10.12	5.90% (1)	88.20% (15)	5.90% (1)
Lipids (g)	29.97 ± 9.76	11.80% (2)	58.80% (10)	29.40% (5)
Iron (mg)	17.7 ± 10.6	23.5% (4)	76.5% (13)	0% (0)
Magnesium (mg)	228.5 ± 86.9	100% (17)	0% (0)	0% (0)
Sodium (mg)	4044.5 ± 2177.6	0% (0)	29.4% (5)	70.6% (12)
Potassium (mg)	2652.9 ± 1131.9	94.1% (16)	5.9% (1)	0% (0)
Phosphorus (mg)	1309.3 ± 689.6	17.6% (3)	82.4% (14)	0% (0)
Calcium (mg)	622.1 ± 386.7	82.4% (14)	17.6% (3)	0% (0)
Selenium (mcg)	24.6 ± 20.1	88.2% (15)	11.8% (2)	0% (0)
Zink (mg)	20.1 ± 14.9	35.3% (6)	64.7% (11)	0% (0)
Vitamin D (mcg)	3.7 ± 4.3	88.2% (15)	11.8% (2)	0% (0)
Vitamin B12 (mcg)	5.4 ± 4.6	29.4% (5)	70.6% (12)	0% (0)
Vitamin A (mcg)	675.5 ± 607.4	76.5% (13)	23.5% (4)	0% (0)
Vitamin C (mg)	109.9 ± 135.3	64.7% (11)	35.3% (6)	0% (0)
Vitamin E (mg)	18.4 ± 10.5	35.3% (6)	64.7% (11)	0% (0)

Descriptive analysis.

*%* percentage, *SD* standard deviation.

Micronutrients were analyzed according to the DRIs^24^ considering the RDA, and it was found that most patients consumed sodium, magnesium, potassium, calcium, selenium, and vitamins D, A, and C outside the normal standards: Sodium consumption was above the recommended range, and the remaining nutrients were below (Table 3).

### Correlations between swallowing aspects, nutritional status, and salivary flow: results

The correlation between swallowing sEMG and anthropometric nutritional assessment showed that the greater the AMC, the shorter the electrical activity contraction time for all studied muscles considering the swallowing of pudding consistency (Table 4).

**Table 4:** Results for the correlation test between the contraction time of the muscles assessed in the sEMG during swallowing of pudding consistency and arm muscle circumference (AMC).

Variables	Swallowing time (s)							
	Left masseter		Right masseter		Suprahyoid muscles		Orbicularis oris	
	*r*	*p*	*r*	*p*	*r*	*p*	*r*	*p*
AMC	− 0.67	0.003*	− 0.73	0.001*	− 0.75	0.001*	− 0.56	0.020*

*p* < 0.05—Spearman's correlation coefficient.

*r* correlation coefficient.

*Statistically significant correlation (*p* < 0.05).

There was no statistical significance for the analysis of the correlation between the findings of the videofluoroscopic swallowing exam with the anthropometric assessment of the nutritional status as well as between the results of the surface electromyography and the videofluoroscopic swallowing exam with the salivary flow with mechanical stimulus.

## Discussion

In this pilot study, the salivary flow test results are consistent with the literature findings that point to patient hyposalivation after head and neck cancer treatment^25^. However, the relationship between the swallowing tests' findings and the salivary flow in this study was not significant probably due to a possible improvement or even an adaptation of the swallowing function.

Regarding, the DOSS scale, 66.7% (8/12) of patients had functional limitations in swallowing (level 6), which is consistent with the work by Costa^26^. This finding can be explained due to the time of post-cancer treatment indicating an improvement in dysphagia, which corroborates studies that indicate an improvement in swallowing in the period from 3 to 12 months after treatment, such as Logemann et al.^27^ and Newman et al.^28^, which points the improvement can happen throughout the 18 months.

The results found on the Penetration-Aspiration Scale indicate that the recovery may take longer for some individuals or that it has more serious consequences due to more aggressive treatment. In this study by the scale of Hind et al.^21^ there was an average of two structures with residues, predominantly vallecula and pyriform sinuses with food residue, the value of which was 1 on average (a line of barium on the evaluated structure) for the three consistencies (pudding, honey, and liquid). No studies using the scale by Hind et al.^21^ with patients after cancer treatment were found. However, studies assessed the presence of residues using other scales, in which residues in valecula and pyriform sinuses were also observed^29^. Such findings can be justified due to radiation injuries from treatment, causing motor, physiological, and sensory alterations that affect the normal swallowing process^30^.

No laryngotracheal aspiration was found, considering the three tested consistencies, which is not similar to the results found in some studies^31,32^. That difference can be justified by the case series, in which the time after treatment of the studies may have been immediate until 7 months after end of treatment. In comparison, this research had an average of 18 months after treatment, as in the research by Pederson et al.^31^ and Hedstrom et al.^32^, which did not consider altered swallowing an inclusion criterion, something that may have contributed to recruiting individuals with better airway protection conditions during swallowing.

The sEMG exam assessed the average electrical activity for the suprahyoid muscles and the average swallowing time. Due to the lack of studies evaluating these parameters in patients after head and neck cancer treatment, the results were compared with studies that described the standardization for electrical activity (µv) and swallowing time (seconds) for healthy individuals^33^. The average electrical activity for the suprahyoid muscles is within normal standards, as well as the average swallowing time for liquid consistency. Pudding and honey consistencies, however, took longer than expected. The parameters assessed in the orbicularis oris muscles and masseters in all consistencies were outside the normal range. One hypothesis for such findings is that patients make compensatory recruitment of other muscle groups due to functional limitations and hyposalivation^34^, ^35^.

Regarding the anthropometric assessment, the small number of participants limited our statistical conclusions. However, patients with below ideal BMI had finished treatment less than 12 months before the study, while those with a BMI above ideal had finished treatment more than 12 months after the study. According to Jager-Wittenaar et al.^36^, patients who had an adequate intake of energy and calories (35 kcal/kg and 1.5 g/protein/kg) daily obtained gain of weight due to the fact that the energy intake is above the ideal and proteins within the recommended. This points out that most patients need time after treatment to regain your lost weight, in which possibly will be able to adapt to their new conditions may lead to a normal BMI or even overweight if they are not well informed about their food choices.

Concerning the assessment of micronutrients evaluated by the average consumption, that indicates that patients made incorrect choices of food, which points to the need for nutritional guidance. Food recall made it possible to analyze that most patients followed a diet of general consistency, with only a few adaptations, such as drinking liquid with the meal to moisten the bolus and help swallowing due to hyposalivation.

Correlating nutritional assessment with electromyographic activity showed that the greater the patients' muscle tissue reserve (AMC), the smaller the amplitude of electrical muscle activity for all muscle groups studied (masseter, suprahyoid, and orbicularis oris) in pudding consistency.

The study's limitations were the variations between the time after head and neck cancer treatment, which varied from 3 to 64 months among participants, in addition to the small number of patients in the sample. Another fact is the limited number of studies that address the proposed theme, making it difficult to compare results.

In conclusion, the present study found a relationship between swallowing function and nutritional status in head and neck cancer survivors: the higher the AMC, the shorter the electrical activity time for all muscle groups studied in pudding consistency. That may indicate that muscle activity during swallowing may be more efficient with the appropriate protein intake. Despite no relationship was found between salivary flow and swallowing function, this pilot study made it possible to expand the knowledge about the relationship between swallowing function and nutritional status in patients after of head and neck cancer treatment, providing subsidies for future studies.

## Methods

This cross-sectional study was approved by the ethics committee of the institution (Universidade de São Paulo) where the research was conducted. Certificate of Presentation for Ethical Consideration (CAAE): 60662616.8.0000.5417. This article confirm that all experiments were performed in accordance with relevant guidelines and regulations and was have been performed in accordance with the Declaration of Helsinki. An average of 390 medical records were analyzed, where 36 were eligible for this study.

They needed to have undergone head and neck cancer treatment at least 3 months prior to the research; be of both genders; be enrolled in the Research Service Center; and have agreed and signed the Informed Consent Form.

Patients undergoing cancer treatment in any body location, with a history of neurological diseases or syndromic conditions, using alternative feeding, in a wheelchair, and/or who had had tracheostomy were excluded.

### Salivary flow test

Salivary flow test was performed with and without mechanical stimulus according to the methodology used by Tarzia^37^, measuring the saliva volume collected using a 5 mL disposable plastic syringe, ignoring the foam. The value was divided by 5, and the result is in mL/minute^37^.

The classification proposed by Flink et al.^9^ was used for the salivary flow volume with and without stimulus.

### Swallowing videofluoroscopy exam

The videofluoroscopy exams were performed with a piece of C-arm system equipment consisting of closed-circuit television, an X-ray generator with an image intensifier, and a video-recording system (Philips BV Libra C-Arm).

Individuals were asked to swallow food as usual. The food was offered in a syringe directly in the patient's mouth, all with Bariogel barium sulfate contrast, in the following consistencies:

- Liquid: 30 mL filtered water, 30 mL contrast added;
- Honey: 35 mL filtered water, 2 g Clight powdered diet grape juice, 25 mL contrast, and 2.25 g Hormel Thick & Easy food thickener powder;
- Pudding: 35 mL filtered water, 2 g Clight powdered diet grape juice, 25 mL contrast, and 4.5 g Hormel Thick & Easy food thickener powder.

### Swallowing dysfunction severity rating: the DOSS scale

After swallowing instrumental assessment, individuals were classified according to their degree of swallowing dysfunction by a speech-language pathologist with training in oropharyngeal dysphagia and experience in analyzing instrumental assessment using the *Dysphagia Outcome and Severity Scale*—DOSS^25^.

### Evaluation of food stasis—residue scale

Instrumental assessment also made it possible to evaluate food stasis after swallowing using the scale proposed by Hind et al.^21^ that evaluates residues in five structures (oral cavity, valecule, posterior pharynx wall (PPW), pyriform sinuses, and upper esophageal sphincter (UES).

### Laryngotracheal penetration-aspiration scale

Laryngeal penetration and laryngotracheal aspiration were classified by the same speech-language pathologist who performed the other swallowing analyses according to the penetration and aspiration scale^22^ for each food offered.

### Swallowing function electromyography assessment

The electromyographic activity was recorded using the Miotec MIOTOOL 400 surface electromyography equipment connected to a laptop via a USB port.

Regarding the electromyographic signal detection and recording, two main aspects influence signal fidelity: signal-to-noise ratio and signal distortion. Both were avoided through a filter for the 20–500 Hz frequencies. Such spectrum allows manual filtering through a band-stop filter to avoid network interference.

Four muscle groups were analyzed during the swallowing assessment: left and right masseter, upper orbicularis oris, and suprahyoid muscles. The ground electrode was placed on the olecranon elbow bone area.

The swallowing process happened under three conditions: usual swallowing of 5 mL food in pudding (20 mL water, 2 g of dietary grape juice, and 2.25 g Hormel Thick & Easy food thickener), honey (26 mL water, 2 g of dietary grape juice, and 2.25 g Hormel Thick & Easy™ food thickener), and liquid (water at room temperature) consistencies.

The test started after 30 s of rest for all consistencies, when then the food was placed into patients' mouth through a syringe. Patients were instructed to hold the food on top of their tongue and swallow it as usual at the speech-language pathologist's signal. Patients were instructed that they could swallow multiple times until all food was gone. Three offers of each consistency were recorded with an interval of 30 s between each.

The signal was shown and interpreted using the Miograph 2.0 software (MIOTEC, São Paulo, Brazil).

### Anthropometric assessment of the nutritional status

Weight, height, arm circumference, and tricipital (TCP), bicipital (BCP), subscapular (SSSF) and suprailiac (SISF) skinfolds were measured. These results made it possible to calculate the body mass index (BMI), arm muscle circumference (AMC), and body fat percentage (%BF).

### Dietary assessment of food consumption

Dietary assessment of food consumption was performed through a 24-h dietary recall showing a detailed register of any food consumed before the survey in a typical day. A suitcase with everyday kitchen utensils (skimmer, ladle, serving spoon, soup spoon, dessert spoon) was used to make it easier for patients to describe how much food they ingested during the interview.

Food consistency was classified according to Garcia et al.^38^ as *general diet* (no food restriction), *soft diet* (foods moistened or cooked for a longer time), *light or pasty diet* (liquid or semi-solid foods such as pureed foods) and *liquid diet* (no-fiber, liquid foods).

Nutritional analysis was performed using the Nutrilife software, and food intake was quantified in terms of calories, macronutrients, and micronutrients. Those values were compared with the reference proposed by the Institute of Medicine of the National Academies—Dietary Reference Intakes (DRIs)^24^, in which the recommended dietary allowances (RDA) values were considered as a reference. In their absence, the adequate intake (AI) was used, and nutrients that exceeded the tolerable upper intake level (UL) value and energy value according to the American Society for Parenteral and Enteral Nutrition (ASPEN)^23^ were considered above the daily intake.

### Statistical analysis

Spearman's correlation coefficient was used to check the correlation between the variables studied.

All statistical procedures were performed using the SPSS v. 26 program with a significance level of 5% (*p* = 0.05).

### Ethical approval and informed consent

This study was approved by the Institutional Review Board (CAAE: 60662616.8.0000.5417), of Bauru School of Dentistry, University of São Paulo. All participants were clearly informed about the research procedures and signed an Informed Consent Form.
